# Regulatory T Cells as a Novel Candidate for Cell-Based Therapy in Kidney Disease

**DOI:** 10.3389/fphys.2020.00621

**Published:** 2020-06-09

**Authors:** Junyu Lu, Jianfeng Zhang, Menghua Chen, Chun Chen, Zhengzhao Li, Pinhu Liao

**Affiliations:** ^1^The First Clinical Medical College of Jinan University, Guangzhou, China; ^2^Intensive Care Unit, The Second Affiliated Hospital of Guangxi Medical University, Nanning, China; ^3^Department of Emergency Medicine, The Second Affiliated Hospital of Guangxi Medical University, Nanning, China; ^4^Department of Cardiology and Endocrinology, The People’s Hospital of Guangxi Zhuang Autonomous Region, Nanning, China; ^5^Department of Emergency Medicine, The First Affiliated Hospital of Guangxi Medical University, Nanning, China

**Keywords:** regulatory T cell, Treg, M2c macrophage, cell-based therapy, kidney injury

## Abstract

Kidney disease is a significant health concern worldwide. Ineffective treatment can lead to disastrous consequences, such as organ failure and death. Research has turned to cell-based therapy, but has yet to produce an effective and reliable treatment for kidney disease. To address this problem, we examined four datasets of gene expression profiles from diseased and healthy kidney tissue in humans, mice, and rats. Differentially expressed genes (DEGs) were screened and subjected to enrichment analyses. Up-regulated genes in diseased kidney tissue were significantly enriched in pathways associated with regulatory T cells (Tregs). Analysis with the xCell tool showed that Tregs were generally increased in diseased kidney tissue in all species. To validate these results *in vivo*, kidneys were removed from mice with Adriamycin-induced nephropathy, and histology confirmed increase of Tregs. Furthermore, Tregs were adoptively transferred from healthy mice into mice with kidney injury, restoring normal structure to the damaged kidneys. Treg cells that were co-cultured with M2c macrophages exhibited up-regulation of chemokine receptors CCR2, CCR5, CCR7, CD62L, and CX3CR1. This may be the mechanism by which M2c cells enhance the migration of Tregs to the site of inflammation. We propose that Tregs may be an effective, novel candidate for cell-based therapy in pre-clinical kidney injury models.

## Introduction

Kidney disease has become a significant global public health problem, with incidence and mortality rates increasing in recent decades (Lentine et al., 2017; Fraser and Roderick, 2019). Renal failure is associated with age (Kazancioglu, 2013) and it affects both men and women (Iseki, 2005). Acute kidney injury (AKI) can increase the risk of chronic kidney disease (CKD), which can eventually lead to end-stage renal disease (Chawla and Kimmel, 2012). The prevalence of AKI is approximately 1.9% in all hospitalized patients and more than 40% in the intensive care unit (Liangos et al., 2006) while the prevalence of CKD is estimated to be 8–16% worldwide (Jha et al., 2013). Though kidney injury is relatively common, there is no specific treatment for it, and most therapies target the underlying disease. Thus, treatment for kidney injury remains a significant challenge for clinicians (Bagshaw and Bellomo, 2007; Jha et al., 2013) and new effective therapeutic strategies are urgently need.

Cell-based regenerative therapy has been extensively evaluated as a treatment for kidney injury in animal models (Fang et al., 2012; Van Koppen et al., 2012; Zhu et al., 2013). Thus far, mesenchymal stem cells and endothelial progenitor cells have been most studied (Papazova et al., 2015). However, a clinical trial giving allogenic mesenchymal stem cells to patients with AKI did not accelerate functional recovery (Swaminathan et al., 2018). Therefore, investigating other candidate cell types is necessary. It is widely recognized that kidney injury is closely related to the immune system and immune cells (Sato and Yanagita, 2018) yet this avenue has not been thoroughly evaluated. We hypothesized that immune cells may be potential candidates for cell-based therapy against kidney injury. Indeed, we show in the present study that the proportion of regulatory T cells (Tregs) was higher in injured kidney tissue than in healthy kidney tissue. We confirmed these findings in mice with Adriamycin-induced nephropathy, suggesting that adoptive transfer of Tregs can improve injury. In addition, we observed *in vitro* that M2c macrophages up-regulate chemokine receptors in Tregs, which may recruit Tregs to sites of inflammation in injured kidney.

## Materials and Methods

### Prediction of Genes Associated With AKI or CKD

We downloaded the following four gene expression datasets from the Gene Expression Omnibus (GEO) database^1^ : (1) GSE12682 (Si et al., 2009) comparing human kidney tissue between 29 healthy controls and 23 CKD samples, measured by the Affymetrix Human Genome U133A 2.0 Array (Affymetrix; Thermo Fisher Scientific, Waltham, MA, United States); (2) GSE12683 (Si et al., 2009) comparing Balb/c mouse kidney tissue between 10 healthy controls and 10 samples with AKI, measured by Affymetrix Mouse Genome 430A 2.0 Array; (3) GSE102513 (Vandenbussche et al., 2018) comparing CD-1 mouse kidney tissue between four healthy controls and four samples CKD induced by Tacrolimus (1 mg/kg/day for 28 days), measured by the Agilent-028005 SurePrint G3 Mouse GE 8x60K Microarray; and (4) GSE85957 (Pavkovic et al., 2014) comparing *Rattus norvegicus* (brown rat) kidney tissue samples between 19 healthy controls and 38 rats with kidney disease induced with 1 or 3 mg/kg cisplatin. In this last dataset, tissue was collected on days 3, 5, 8, and 26, and gene expression was measured using the Rat Genome 230 2.0 Array.

If one gene matched multiple probes, the average value of all probes was calculated as the expression of the corresponding gene. All sample collection and experimentation procedures were approved by the Guangxi Medical University Ethics Committee.

### Screening of Differentially Expressed Genes (DEGs) and Functional Enrichment Analysis

Differentially expressed genes (DEGs) between healthy control and diseased human kidney tissue were identified using the *limma* package (Ritchie et al., 2015) in R from dataset GSE12682. Differential threshold was calculated based on two criteria: false discovery rate (FDR) *P*-value < 0.05 and | log_2_(fold change)| > 1. The *ClusterProfiler* package (Yu et al., 2012) in R was used to assess enrichment in biological processes (BPs) and Kyoto Encyclopedia of Genes and Genomes (KEGG) pathways. Gene set enrichment was analyzed using the JAVA program^2^ based on MSigDB immunologic signatures (*c7.all.v6.2.symbols.gmt*) (Godec et al., 2016). Gene sets with a NOM *P*-value < 0.05 after performing 1000 permutations were considered to be significantly enriched (Subramanian et al., 2005).

### Immune Cell Enrichment Analysis

We used xCell web tool^3^ (Aran et al., 2017) with Charoentong signature (Charoentong et al., 2017) to generate cell enrichment scores based on gene signature profiles. Then these data were used to quantify immune cell proportions in all four datasets. Enrichment scores were compared using Student’s *t*-test, and *P* < 0.05 was considered to indicate a significant difference.

### Mouse Model of Kidney Injury

Animal experiments were approved by the Guangxi Medical University Ethics Committee. C57BL/six mice (8 weeks old, 20 ± 2 g) were purchased from the Experimental Animal Center of Guangxi Medical University. Mice were randomly divided into a control group (*n* = 27) and a treatment group (*n* = 27). The treatment group received 10.5 mg/kg Adriamycin (Zhejiang Hisun Pharmaceutical, Taizhou, China) three times per week for 2 weeks via the tail vein (Lu et al., 2013). The control group received injections of physiological saline via the tail vein. Urine was collected for 16 h overnight on day 27 for measurement of urinary protein. Blood was obtained on day 28 for measurement of serum creatinine and creatinine clearance. All urine and blood specimens were analyzed using automated analyzers at the Experimental Animal Center of Guangxi Medical University. Animals were sacrificed using cervical dislocation on day 28, and kidneys and spleens were removed for further study.

### Preparation of Cell Suspensions

Kidneys were perfused with saline before removal, cut into 1–2 mm^3^ pieces and digested in Dulbecco’s modified Eagle medium (DMEM) containing 1 mg/ml collagenase IV (Sigma–Aldrich) and 100 mg/ml DNase I (Roche) for 40 min at 37°C with intermittent agitation. The digested cell suspension was then passed through a 40-μm cell strainer. Mononuclear cells were separated using 1.077 g/ml NycoPrep gradient (Axis-Shield, Oslo, Norway). Spleens from C57BL/6 mice were also isolated, minced, and digested for 30 min at 37°C in RPMI 1640 containing 1 mg/ml collagenase D and 100 mg/ml DNase I (both from Roche). The digested cell suspension was passed through a 40-μm cell strainer.

### Flow Cytometry and Cell Sorting

Single-cell suspensions from kidney were subjected to magnetic bead separation, and CD3+ cells were purified using the CD3+ T cell kit (Miltenyi Biotec, Bergisch Gladbach, Germany). The surface of CD3+ cells was labeled with antibodies against CD25 and FOXP3 (BD Accuri C6, United States). Double-positive cells were considered Tregs and were sorted using a flow cytometer (BD Accuri C6, United States) according to the manufacturer’s instructions.

### Mouse Model of Adoptive Cell Transfer

Tregs were extracted from spleens of healthy C57BL/six mice, and single-cell suspensions were prepared as in Section “Preparation of Cell Suspensions.” Cells were incubated *in vitro* in RPMI 1640 medium and allowed to proliferate for 7 days. Approximately 1.2 × 10^6^ Tregs were injected via the tail vein into mice on day 5 after Adriamycin administration.

### Tissue Histology and Immunofluorescence

All mice were sacrificed on day 28. Coronal sections of renal tissue were stained with rat anti-mouse CD103 antibody (M290, 1:200) and hamster anti-mouse CD11c antibody (N418, 1:100), then incubated with AF488-conjugated goat anti-rat IgG or AF546-conjugated goat anti-hamster IgG antibody (both 1:500). Tissue sections were analyzed on an inverted fluorescence microscope (BX50, Olympus). Consecutive sections from kidney cortex to kidney medulla were photographed.

### *In vitro* Co-cultures

Bone marrow from the femurs of C57BL/6 mice was harvested and triturated with sterile syringes, and the resulting cell suspension was filtered through 40-μm nylon mesh. Approximately 2 × 10^6^ cells were incubated in RPMI 1640 containing 50 ng/ml granulocyte-macrophage colony stimulating factor (GM-CSF) at 37°C for 10 days. The adherent marrow-derived macrophages (M0) were rinsed three times in RPMI 1640 medium and then incubated in RPMI 1640 medium containing both IL-10 and TGF-β (10 ng/ml each) for 4 days to become M2c macrophages (Lu et al., 2013).

For co-culture experiments, M2c macrophages (1 × 10^6^ cells/ml) were plated in six-well plates with 1.5 ml of Tregs (1 × 10^6^ cells/ml). Co-cultures were incubated for 24 h at 37°C and 5% CO_2_. Control cultures contained only Tregs. After 24 h, the floating Tregs were collected, washed in RPMI 1640, and used in subsequent experiments.

#### PCR Array and Quantitative PCR (qPCR)

Total RNA was extracted from control and co-cultured Tregs using the TRIzol RNeasy kit (Invitrogen, Shanghai, China), then reverse-transcribed using the Superscript IV reverse transcription Kit (Invitrogen). Quantitative PCR was performed using the SYBR qPCR (Invitrogen) master mix according to the manufacturer’s instructions. The primer list is shown in Supplementary Table S1.

#### Western Blotting

Total protein was extracted from control and co-cultured Tregs using 200 μL ice-cold RIPA lysis buffer (catalog no. R0010, Solarbio, Beijing, China) at 4°C for 2 min and centrifuged. The supernatant was collected, protein concentration was estimated using a BCA kit (Solarbio, Beijing, China), and an equal amount of total protein in each sample was mixed with 5 μl of 5× SDS buffer and 2 μl of reducing agent (Invitrogen), then boiled at 70°C for 10 min. Samples were loaded onto a sodium dodecyl sulfate-polyacrylamide gel (8–12% gradient), then transferred onto polyvinylidene fluoride membranes. Membranes were immunoblotted using antibodies (Abcam, Cambridge, United Kingdom) against the following proteins: GADPH (catalog no. ab9484), CCR2 (ab203128), CCR7 (ab32527), CCR8 (ab63772), CD62L (ab264045), and CX3CR1 (ab8021). The secondary antibody was horseradish peroxidase-conjugated goat anti-rabbit IgG H&L (ab205718). GADPH protein was used as the internal control. Bands were developed using the ECL Western Blotting Analysis System (Amersham, Sweden) and visualized with FluorChem E ProteinSimple (ProteinSimple, United States).

### Statistical Analysis

Statistical tests included the unpaired, two-tailed *t*-test using Welch’s correction for unequal variances and one-way ANOVA with Tukey’s multiple comparisons test. Statistical analyses were performed using Prism software (version 5, GraphPad) and R software (version 3.5.3). Results were expressed as mean ± SEM. Differences associated with *P* < 0.05 were considered statistically significant.

## Results

**FIGURE 1:**
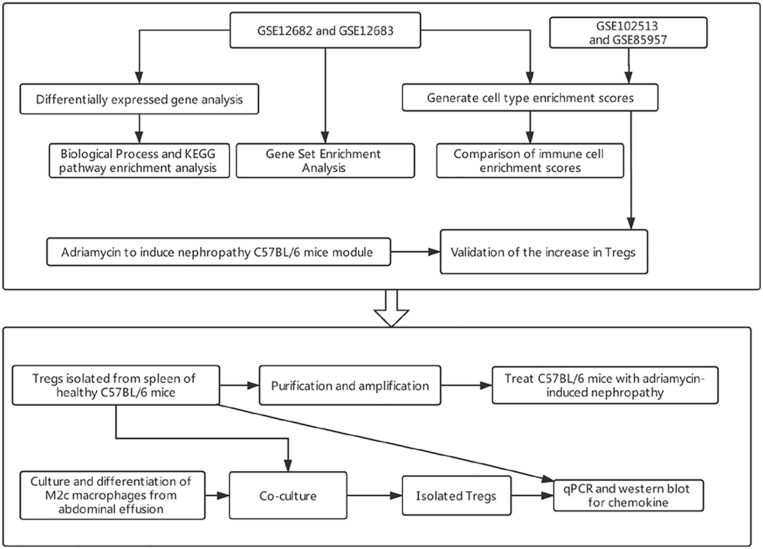
The work flow of the present study. KEGG, Kyoto Encyclopedia of Genes and Genomes.

The study workflow is presented in Figure 1.

### Immune System-Related Genes Are Up-Regulated After Kidney Injury

**FIGURE 2:**
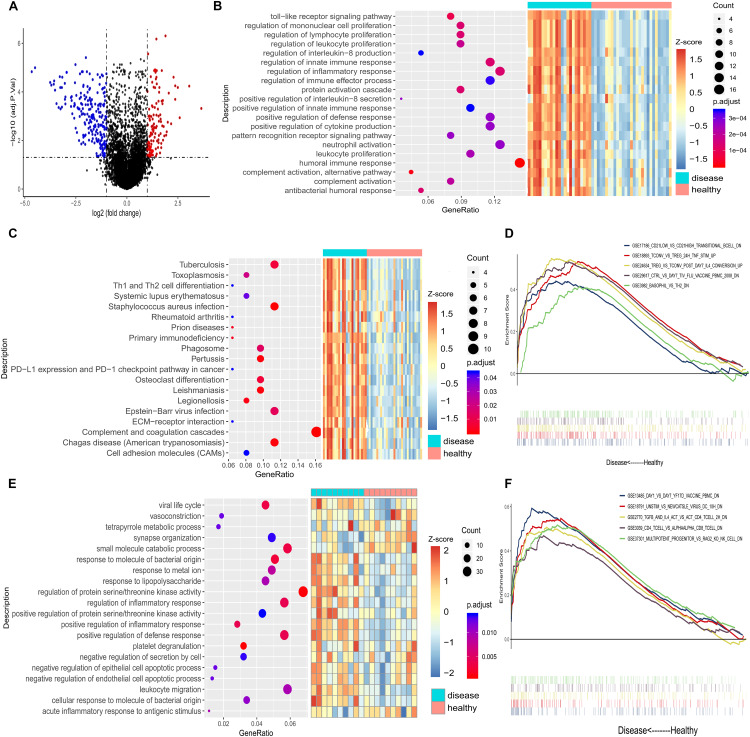
**(A)** Volcano plot of the genes differentially expressed between chronic kidney disease tissue and healthy kidney tissue from humans (dataset GSE12682). Red indicates that a gene is up-regulated in chronic kidney disease, while blue indicates that it is down-regulated. **(B)** The 15 most significantly enriched biological processes from the up-regulated genes in GSE12682. **(C)** Enriched KEGG pathways for the up-regulated genes in GSE12682. **(D)** The five most significantly enriched gene sets in chronic kidney disease in GSE12682. **(E)** The 15 most significantly enriched biological processes for the up-regulated genes from Balb/c mice (dataset GSE12683). **(F)** The five most significantly enriched gene sets in Adriamycin-induced kidney injury in GSE12683.

In human kidney tissue (GSE12682), we found a total of 345 DEGs by comparing healthy control tissue with CKD tissue (Figure 2A), of which 123 genes were up-regulated and 222 down-regulated. The down-regulated genes were enriched in various renal system development-related BPs and KEGG pathways (Supplementary Tables S2, S3), whereas up-regulated genes were immune-related (Figures 2B,C). Although no DEGs were identified in Balb/c mice (GSE12683) using the initial threshold (FDR *P* < 0.05 and | log_2_(fold change)| > 1), using a less stringent threshold (*P* < 0.05) identified 660. These included 330 up-regulated genes enriched in various immune-related BPs (Figure 2E). These results suggest that genes of the immune system are activated in injured kidney, and that identification of immune cells associated with these up-regulated genes may provide candidate therapeutic targets against kidney injury.

### Tregs Enriched in Injured Kidney

**FIGURE 3:**
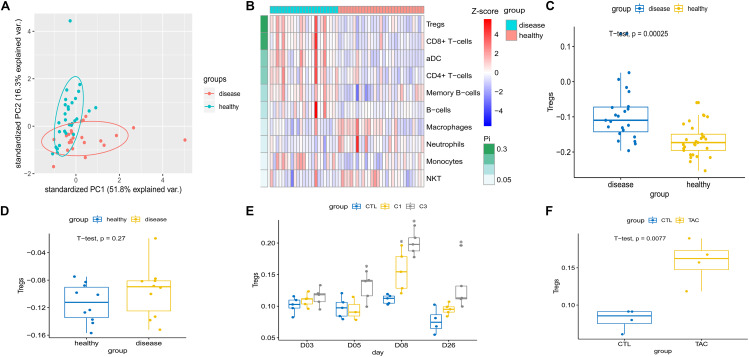
**(A)** Principal component analysis showed a significant difference in the immune cell population between chronic kidney disease tissue and healthy kidney tissue in humans (dataset GSE12682). **(B)** Heatmap depicting abundance of different immune cell types between chronic kidney disease and healthy kidney in GSE12682. Enrichment analysis of Treg-related genes was calculated and numbers of cells extrapolated for **(C)** chronic kidney disease compared to healthy kidney in humans (GSE12682), **(D)** Adriamycin-induced kidney injury compared to healthy kidney in Balb/c mice (GSE12683), and **(E)** cisplatin-induced kidney injury compared to healthy kidney in rat (GSE85957). *P < 0.05 versus control. **(F)** Tacrolimus-induced kidney injury compared to healthy kidney in CD-1 mice (GSE102513). C1, 1 mg/kg cisplatin; C3, 3 mg/kg cisplatin; var., variance; CTL, control; TAC, tacrolimus.

Gene set enrichment analysis confirmed that among the up-regulated immune-associated gene sets, those associated with Tregs were significantly enriched in both human CKD tissues (Figure 2D) and mouse AKI tissues (Figure 2F). Extraction of genetic profiles associated with different immune cells from all datasets and scoring them for enrichment (Figure 3A and Supplementary Table S4) further confirmed Tregs to be more abundant in diseased kidney tissue than in healthy control tissue from humans, mice, or rats, regardless of the cause of tissue injury (Figures 3B–F).

### Tregs Are Increased in Kidneys From Adriamycin-Induced Injury Mouse Models

**FIGURE 4:**
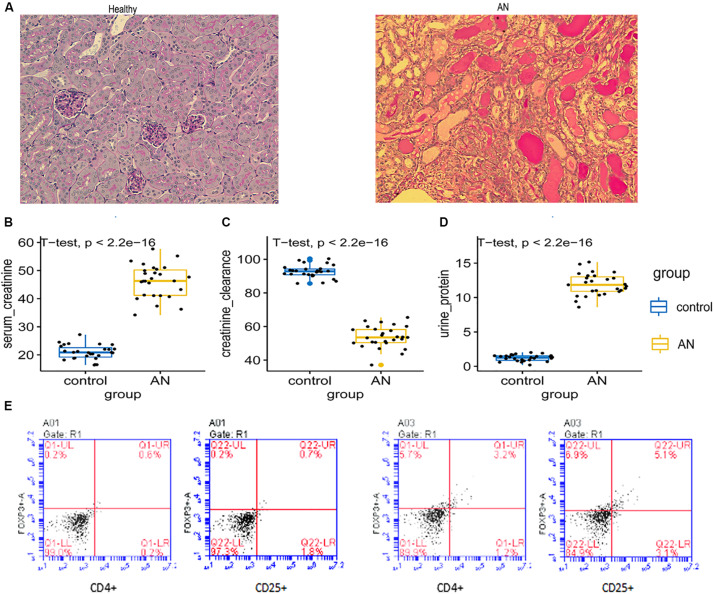
**(A)** Histology of kidney with Adriamycin-induced nephropathy showed severe structural injury, in contrast to healthy control kidney. **(B–D)** Serum creatinine, urine protein, and creatinine clearance were assessed in control mice and mice with Adriamycin-induced nephropathy. **(E)** Dot plots from flow cytometry showing numbers of CD4+ CD25+ Tregs isolated from control kidney and kidney with Adriamycin-induced nephropathy. Percentages of cells are indicated within each quadrant. LL, lower left; LR, lower right; Q, quadrant; UL, upper left; UR, upper right.

Histology confirmed Adriamycin-induced structural kidney injury in mice (Figure 4A), including glomerular sclerosis, tubular atrophy, and interstitial expansion. Severe functional injury was indicated by increased serum creatinine (Figure 4B) and proteinuria (Figure 4C), as well as reduced creatinine clearance (Figure 4D). Numbers of CD4+ CD25+ Tregs were significantly higher in Adriamycin-injured kidneys than in control kidneys (5.1 versus 0.7%) (Figure 4E).

### Tregs Can Protect Against Induced Kidney Injury

**FIGURE 5:**
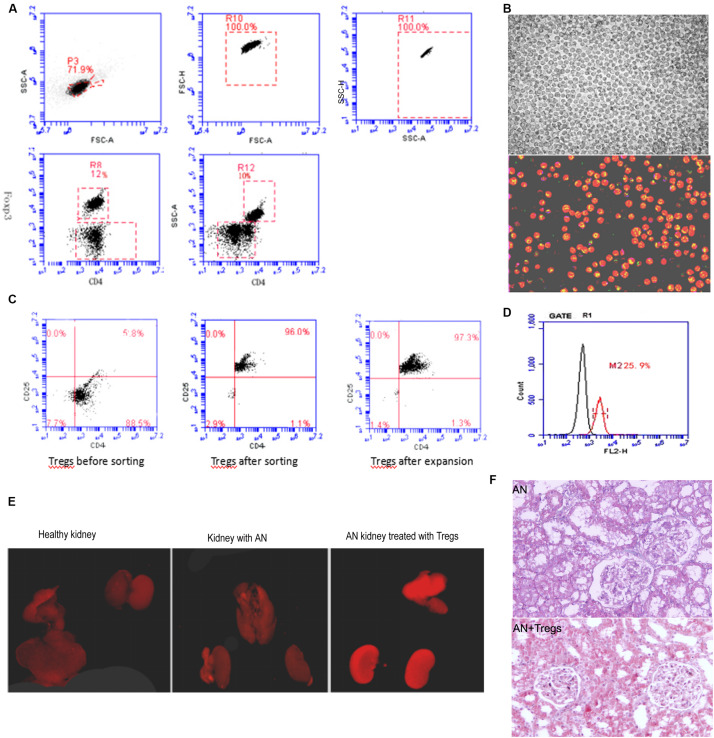
**(A)** Dot plots from flow cytometry showing numbers of CD4+ CD25+ Tregs isolated from spleens of healthy mice. **(B)** Increase in Treg numbers after *in vitro* amplification. **(C)** Dot plots from flow cytometry showing purity of CD4+ CD25+ Tregs after amplification. **(D)** Flow cytometry histogram depicting Treg numbers isolated from mice with Adriamycin-induced nephropathy in which Tregs from healthy donors had been adoptively transferred. **(E)** Fluorescence images of kidneys from control mice, mice with Adriamycin-induced nephropathy, and mice with nephropathy after treatment with Tregs from healthy donors. **(F)** Histological examination suggested that kidney with AN treated with Tregs show less damage than AN. AN, Adriamycin-induced nephropathy.

To examine whether the influx of Tregs mitigates or aggravates pre-existing kidney damage, we used a mouse model of adoptive cell transfer. CD4+ CD25+ Tregs were isolated from spleens of healthy mice and expanded *in vitro* (Figures 5A,B). Treg cultures of 90% purity were injected via the tail vein into mice with Adriamycin-induced kidney injury (Figure 5C). Kidneys were isolated on day 28 after Adriamycin administration and histology revealed that large numbers of Tregs had been recruited to the injured kidney, such that Tregs were more abundant in injured kidney than in healthy kidney (Figure 5D). Fluorescence imaging (Figure 5E) and histological imaging (Figure 5F) showed that adoptive transfer of Tregs aided recovery of structural defects in the kidney injury model.

### M2c Macrophages May Enhance the Therapeutic Effects of Tregs

**FIGURE 6:**
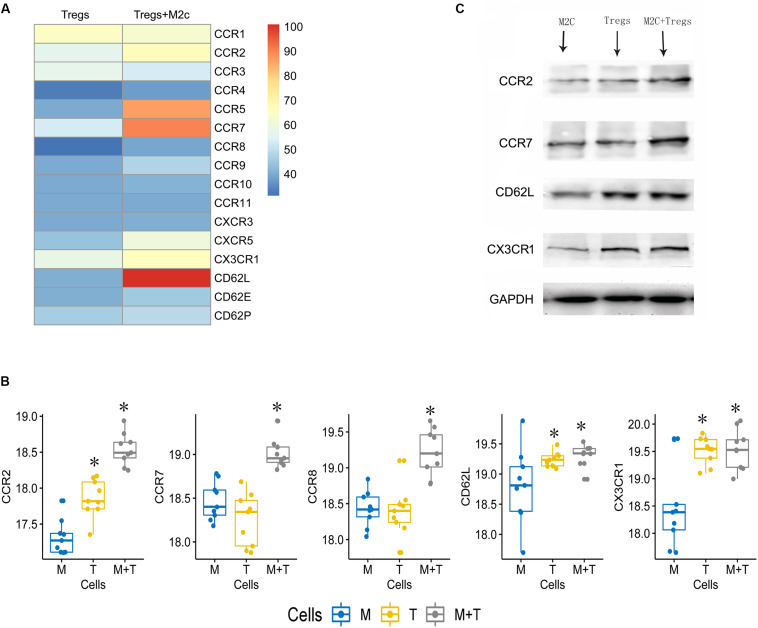
**(A)** Heatmap showing expression of 16 chemokines in Tregs cultured alone or together with M2c macrophages. **(B,C)** Western blot validation of the chemokine up-regulation observed in panel. M, M2c macrophages; T, regulatory T cells. *P < 0.05 versus M2c macrophages.

M2c macrophages may induce Tregs to prevent Adriamycin-induced injury in mice (Kinsey et al., 2012). Indeed, we found that Tregs co-cultured with M2c macrophages showed significant up-regulation of chemokines CCR2, CCR5, CCR7, CD62L, CX3CR1 at the mRNA level (Figures 6A–C) and protein level (Figures 6B,C). M2c macrophages may promote the protective effects of Tregs in kidney by increasing expression of these chemokines.

## Discussion

Despite the global health burden due to kidney injury, treatments are seriously lacking. In end-stage renal failure, patients require a kidney transplant. However, because of shortages in donor organs, most of these patients rely on hemodialysis—but even this intervention has limited success in improving survival (Lameire et al., 2009; Ortiz et al., 2014). In recent years, cell-based therapy in various animal models has shown promise for slowing the progression of kidney disease (Togel and Westenfelder, 2012). In the present study, we found that genes up-regulated in injured kidney tissue are significantly enriched in immune-related BPs and pathways, especially those associated with Tregs. This suggests that the immune system is activated after kidney injury. Tregs were generally increased in injured kidney tissue, including CKD samples from humans (GSE12682) and mice (GSE102513), AKI samples from mice (GSE12682), and CKD samples from rat (GSE85957). We were unable to find publicly available gene expression profiles of Adriamycin-induced kidney injury. Then we validated the increase of Tregs in mice with Adriamycin-induced kidney injury, a model we selected because of its usefulness in studying the pathogenesis and progression of this condition (Lu et al., 2013; Cao et al., 2016) and because it involves inflammatory reactions that mimic those in both AKI and CKD (Chawla and Kimmel, 2012; Coca et al., 2012). We further showed that adoptive Treg transfer from healthy mice into Adriamycin-treated animals protected against kidney injury. In addition, we found evidence that M2c macrophages work synergistically with Tregs to combat kidney injury by enhancing expression of chemokines in Tregs, which likely recruits more Tregs to sites of inflammation (Zhang et al., 2009).

A previous meta-analysis study indicated that cell-based therapies improved renal function and morphology in preclinical models of CKD (Papazova et al., 2015). Unlike our study, that meta-analysis included mesenchymal stem cells, endothelial progenitor cells, hematopoietic stem cells, and bone marrow cells (Papazova et al., 2015) so the included studies involved a diversity of disease models. Mesenchymal stem cell-based therapy has been shown to improve renal function and recovery of damaged renal tissues in animal studies (Yun and Lee, 2019), but it has yet to produce strong positive results in clinical trials (Yun and Lee, 2019). Most clinical studies using hematopoietic stem cell therapy have focused on lupus nephritis and have lacked a control group or randomization (El-Ansary et al., 2012; Alchi et al., 2013). The present study shows, through systematic analysis of different types of kidney injury in different species, that harnessing Tregs may be a strategy to treat kidney disease. We validated this hypothesis using mice with Adriamycin-induced kidney injury. Future work should identify whether other types of immune cells than Tregs also inhibit abnormal immune processes and promote kidney repair (Dong et al., 2019). If so, combining Tregs with these other cell type(s) may be an even more effective treatment.

Indeed, our own previous work showed that alternatively activated macrophages (M2 phenotype), including M2a and M2c subpopulations, exhibit anti-inflammatory functions *in vitro* and protect against renal injury *in vivo* (Wang et al., 2007). M2c macrophages appear to protect against renal injury better than M2a macrophages because of their ability to induce Tregs (Lu et al., 2013). We provide evidence here that M2c macrophages may increase CCR2, CCR5, CCR7, CD62L, and CX3CR1 expression in Tregs. This may be one of the mechanisms by which M2c cells recruit Tregs. Future work should explore whether these macrophages can promote the efficacy of Treg-based kidney therapies.

Although the current study provides data supporting a novel cell-based treatment of kidney injury, it has some limitations. First, we assessed the effects of Tregs on injured kidney purely in morphological terms, so future work should seek to explain the molecular basis of this structural recovery, such has by identifying changes in gene expression, particularly of genes involved in fibrosis. Second, we did not begin to assess the safety of adoptive Treg transfer; future work should verify and extend our adoptive transfer studies by including control animals treated with other cell types such as red blood cells. We suspect that the anti-inflammatory Tregs may induce adverse effects similar to those of immunosuppressive drugs. Those studies should also test Treg therapy in other models of kidney injury.

## Conclusion

Tregs are a potential candidate for cell-based treatment of kidney disease. The relationship between M2c macrophages and Tregs should be explored as a potential way to increase therapeutic efficacy.

## Data Availability Statement

All datasets presented in this study are included in the article/Supplementary Material.

## Ethics Statement

The animal study was reviewed and approved by Guangxi Medical University.

## Author Contributions

JL and JZ designed and coordinated the study and prepared the manuscript. MC and ZL provided assistance in the design of the study and participated in manuscript preparation. CC and PL participated in data gathering. All authors have read and approved the content of the manuscript.

## Conflict of Interest

The authors declare that the research was conducted in the absence of any commercial or financial relationships that could be construed as a potential conflict of interest.

## Footnotes

1 www.ncbi.nlm.nih.gov/geo

2 http://www.broadinstitute.org/gsea

3 http://xcell.ucsf.edu/
